# Cellular Mechanisms of Etrolizumab Treatment in Inflammatory Bowel Disease

**DOI:** 10.3389/fphar.2019.00039

**Published:** 2019-02-01

**Authors:** Charlotte Lichnog, Sha Klabunde, Emily Becker, Franklin Fuh, Philipp Tripal, Raja Atreya, Entcho Klenske, Rich Erickson, Henry Chiu, Chae Reed, Shan Chung, Clemens Neufert, Imke Atreya, Jacqueline McBride, Markus F. Neurath, Sebastian Zundler

**Affiliations:** ^1^Department of Medicine 1, Friedrich-Alexander-Universität Erlangen-Nürnberg, Kussmaul Campus for Medical Research and Translational Research Center, Erlangen, Germany; ^2^OMNI Biomarker Development, Development Sciences, Genentech, Inc., South San Francisco, CA, United States; ^3^Optical Imaging Centre, Friedrich-Alexander-Universität Erlangen-Nürnberg, Erlangen, Germany; ^4^BioAnalytical Sciences, Development Sciences, Genentech, Inc., South San Francisco, CA, United States; ^5^Biochemical and Cellular Pharmacology, Genentech, Inc., South San Francisco, CA, United States

**Keywords:** etrolizumab, inflammatory bowel diseases, internalization, STED microscopy, adhesion

## Abstract

**Background:** Anti-integrin therapy is a new frontline strategy in the treatment of inflammatory bowel diseases (IBD). The anti-β7 integrin antibody etrolizumab is currently being investigated for safety and efficacy in Crohn’s disease (CD) and ulcerative colitis (UC) in several phase III trials. Mechanistically, etrolizumab is known to block β7 integrin ligand binding and reduces intestinal trafficking of β7-expressing cells. Etrolizumab blocks β7 integrin ligand binding and reduces β7-positive lymphocyte migration and retention in the inflamed gut mucosa, but the exact mechanisms by which this inhibition occurs are not fully understood.

**Methods:** Cellular effects of etrolizumab or etrolizumab surrogate antibody (etrolizumab-s) were investigated in cell culture models and analyzed by flow cytometry, fluorescence microscopy, ImageStream^®^, stimulated emission depletion (STED) microscopy and functional dynamic *in vitro* adhesion assays. Moreover, effects on α4β7 integrin were compared with the pharmacodynamically similar antibody vedolizumab.

**Results:** As demonstrated by several different approaches, etrolizumab and etrolizumab-s treatment led to internalization of β7 integrin. This resulted in impaired dynamic adhesion to MAdCAM-1. Internalized β7 integrin localized in endosomes and re-expression of β7 was dependent on *de novo* protein synthesis. *In vitro* etrolizumab treatment did not lead to cellular activation or cytokine secretion and did not induce cytotoxicity. Internalization of α4β7 integrin was increased with etrolizumab compared with vedolizumab.

**Discussion:** Our data suggest that etrolizumab does not elicit secondary effector functions on the single cell level. Integrin internalization may be an important mechanism of action of etrolizumab, which might explain some but not all immunological effects observed with etrolizumab.

## Introduction

Inflammatory bowel diseases (IBD), such as Crohn’s disease (CD) and ulcerative colitis (UC) are marked by intestinal immune cell infiltration leading to pro-inflammatory signaling and tissue destruction (Strober et al., 2007; Kaser et al., 2010; Neurath, 2014). Such cell accumulation in the gut is controlled by cell trafficking processes including gut homing and intestinal retention (Habtezion et al., 2016; Zundler and Neurath, 2017). Adhesion of lymphocytes dependent on activated α4β7 integrin to mucosal vascular addressin cell adhesion molecule (MAdCAM)-1 expressed on high-endothelial venules in the gut has been identified as an important mechanism of gut homing (Berlin et al., 1993; Zundler et al., 2017a). The translational potential of this mechanism has been impressively demonstrated by the successful clinical implementation of inhibiting the α4β7 integrin by the monoclonal antibody vedolizumab as a therapeutic strategy in IBD (Feagan et al., 2013; Sandborn et al., 2013). In addition to α4, the β7 integrin monomer also pairs with αE to form the αEβ7 heterodimer, which has been shown to control epithelial retention of homed lymphocytes in intestinal inflammation (Cepek et al., 1994). The anti-β7 antibody etrolizumab is currently being investigated in several phase III trials in IBD patients and blocks both α4β7-mediated gut homing as well as αEβ7-controlled retention (Vermeire et al., 2014; Zundler et al., 2017c).

While these mechanisms have been proposed by cell trafficking studies (Zundler et al., 2017c), the molecular mechanisms responsible for the effect of etrolizumab have so far not been described. Here, we addressed the hypothesis derived from previous studies with vedolizumab (Wyant et al., 2013) that one mechanism of action of etrolizumab might be internalization of β7 integrin leading to unavailability of the integrin on the cell surface. We show with complementary techniques including molecular microscopy with stimulated emission depletion (STED) imaging and ImageStream^®^ analyses that etrolizumab leads to internalization of α4β7 integrin, that this functionally impairs β7-dependent adhesion to MAdCAM-1, and that internalization of α4β7 integrin is higher with etrolizumab compared with vedolizumab.

## Materials and Methods

### Patients With IBD

Peripheral blood was collected from patients with CD (*n* = 53) and UC (*n* = 44) following prior informed written consent at the Outpatient Department of the Medical Clinic 1 of the University Hospital Erlangen. Control blood was obtained from healthy donors (*n* = 27). Clinical data of blood donors are summarized in Table 1. Blood collection was approved by the Ethics committee of the Friedrich-Alexander University Erlangen-Nuremberg. For some experiments, peripheral blood samples were collected from an anonymous internal Genentech blood donor program of healthy volunteers.

**Table 1:** Patient characteristics.

		CON	CD	UC
Number		27	53	44
Age [years] (Mean +/- SEM)		26 +/- 1	37 +/- 2	41 +/- 2
Female [%]		63	41	41
HBI (Mean +/- SEM)			4.0 +/- 0.4	
PMS (Mean +/- SEM)				1.7 +/- 0.3
Concomitant therapy [%]	Immunosuppressants		11.3	4.6
	Steroids		1.9	0
	Mesalazin		0	6.8
	Vedolizumab		0	0
	Anti-TNF antibodies		86.8	88.6
Localization [%]			L1: 17.0	E1: 18.6
			L2: 11.3	E2: 32.6
			L3: 41.5	E3: 48.8
			L4: 0	
			L4+: 30.2	

*HBI, Harvey-Bradshaw Index; PMS, Partial Mayo Score.*

### Cell Isolation

Peripheral blood mononuclear cells (PBMCs) were isolated by standard density gradient centrifugation with either Lymphocyte Separation Buffer (Anprotec) or Ficoll Paque (GE). Where indicated, CD4^+^ T cells were purified from PBMCs with CD4 microbeads (Miltenyi).

### Etrolizumab Surrogate Antibody (Etrolizumab-s) Internalization

For assessment of etrolizumab-s internalization, PBMCs were treated with 10 μg/mL of the etrolizumab surrogate rat antibody FIB504 (Genentech) in phosphate buffered saline (PBS) or in RPMI 1640 medium (Thermo Fisher) with 1% penicillin/streptomycin (Biochrom) and 10% FCS (Pan Biotech) at 4°C and/or 37°C for 24 h. Etrolizumab-s is the parent antibody of etrolizumab (Stefanich et al., 2011). For some experiments, etrolizumab-s was labeled with AlexaFluor (AF) 647 with an AF647 labeling kit (Thermo Fisher) according to the manufacturer’s instructions.

Acid wash was performed with a solution of 0,5M NaCl + 0,2M acetic acid as previously described (Wyant et al., 2013).

### Assessment of Etrolizumab Internalization With ImageStream^®^

Peripheral blood mononuclear cells were treated with AF488-labeled etrolizumab (10 μg/mL) for 24 h at 37 or 4°C. Cells were washed and stained with CD4 and CD45RA, and subsequently with or without a quenching anti-AF488 antibody (addition of 25 μg to cell pellet on ice for 1 h, Thermo Fisher Scientific). Surface and intracellular fluorescence signals were recorded using the ImageStream^®^X Mark II Imaging Flow Cytometer (MilliporeSigma).

### Flow Cytometry

The following antibodies were used for cell staining according to standard flow cytometry protocols: CD4 (VIT4, VioBlue/FITC, Miltenyi; RPA-T4, PE/V450, BD Biosciences), CD8 (RPA-T8, PE-Cy7, BD Biosciences), CD45RA (HI100, V500/APC/BV510, BD Biosciences/Biolegend), CD19 (SJ25C1, APC-Cy7, BD Biosciences), IgD (IA6-2, V450, BD Biosciences), CD25 (BC96, BV510, Biolegend), CD69 (FN50, APC/Cy7, Biolegend), CD103 (αE integrin) (Ber-ACT8, FITC/APC, BD Biosciences), IFN-γ (B27, PE/Cy7, Biolegend), IL-4 (8D4-8, AF488, Biolegend), IL-17A (BL168, PE, Biolegend), IL-9 (MH9A4, AF647, Biolegend), CD49d (α4) (9F10, FITC/APC, BD Biosciences), 7AAD (BD Biosciences). Additionally, etrolizumab, the anti-β7 integrin antibody FIB504 and the anti-α4β7 integrin antibody Act-1 (all Genentech) labeled with AF647 (AF647 labeling kit, Thermo Fisher) and etrolizumab and the anti-β7 integrin antibody 9D8 (Genentech) labeled with AF488 (AF488 labeling kit, Thermo Fisher) were used.

Where applicable, cells were fixed and permeabilized with the Foxp3 fixation and permeabilization kit (Thermo Fisher).

For analysis of cellular activation (Wyant et al., 2013) upon etrolizumab-s treatment, cells were treated with 1 μg/mL etrolizumab-s for 6 h. Untreated PBMCs cultured for 6 h were used as negative control, while PBMCs treated with 50 ng/mL PMA (Sigma) and 1 μM ionomycin (Cayman) for 6 h served as positive control. After 2 h of culture, all cells were treated with 10 ng/μL Brefeldin A (Applichem) for the remaining 4 h.

For the analysis of β7 integrin re-expression, baseline expression of β7 was determined in PBMCs. Subsequently, these PBMCs were cultured in the presence of etrolizumab-s at 37°C for 24 h. Next, cells were harvested, washed and then re-seeded in cell culture plates for a further 96 h in the presence or absence of Brefeldin A. The time course of β7 integrin re-expression was determined by flow cytometric analysis of aliquots cells harvested at 24, 48, 72, and 120 h from baseline.

Flow cytometry was performed on MACSQuant X Analyzer (Miltenyi) and BD FACSCanto^TM^ II (BD Biosciences) instruments. Data were analyzed with BD FACSDiva Software v8.0. and FlowJo v7.6.5 and v10.1.

### Comparison of Etrolizumab- and Vedolizumab-Induced α4β7 Internalization

Peripheral blood mononuclear cells were pre-incubated with saturating concentrations of unlabeled etrolizumab and vedolizumab at 4°C for 2 h. Cells were then washed and incubated at 4 or 37°C for 24 h prior to a subsequent wash and staining with the non-competing anti-β7 monoclonocal antibody 9D8-AF488. Surface β7 expression was assessed by flow cytometry in subsets of B and T lymphocytes.

### Dynamic *in vitro* Adhesion Assay

Peripheral blood mononuclear cells were cultured for 24 h at 37°C in the presence or absence of etrolizumab-s. Next, cells were labeled with carboxyfluorescein succinimidyl ester (CFSE; Life Technologies). Suspensions of 1.5 million cells/mL in adhesion buffer (pH 7.4, 150 mM NaCl, 10 mM HEPES, 1 mM CaCl_2_, 1 mM MgCl_2_, 1 mM MnCl_2_) were prepared and etrolizumab-s was added or not to aliquots of so far untreated cells. Capillaries for dynamic *in vitro* adhesion assays were prepared as previously described (Binder et al., 2018). In brief, miniature borosilicate capillaries (Vitrocom) were coated with 5 μg/mL rhMAdCAM-1-Fc-chimera (R&D Systems) in 150 mM NaCl with 10 mM HEPES for 1 h at 37°C. Next, unspecific binding sites were blocked with 5% bovine serum albumine (BSA) in phosphate buffered saline (PBS) for 1 h at 37°C.

Perfusion was performed with a peristaltic pump (Shenchen LabV3) at a flow rate of 10 μL/min. Dynamic adhesion was analyzed with time-lapse confocal microscopy (Leica SP8) over 3 min and analyzes with ImageJ (NIH) as previously described (Binder et al., 2018).

### Immunofluorescence

Peripheral blood mononuclear cells were treated with AF647-labeled etrolizumab-s for 24 h at 37 or 4°C. In some experiments, cells were permeabilized with 0.1 % Triton X (Roth) after etrolizumab-s incubation and additionally stained with LAMP-1 (H4A3, AF488, Biolegend) or EEA (5632C2, AF488, Novus Bio) to visualize lysosomes and endosomes, respectively. Subsequently, cells were counterstained with Hoechst dye, suspended in Mowiol (Roth) and covered on microscopy slides. Analyses were performed with fluorescence microscopy (Leica DM6000B). Surface and intracellular fluorescence signals were quantified with ImageJ (NIH) by determining the mean fluorescence intensity (MFI) of regions of interest defined around or in projection to the nuclei, respectively.

### STED-Microscopy

To increase the number of β7 integrin-expressing cells, PBMCs were stimulated with anti-CD3 (OKT3, eBioscience) and anti-CD28 antibodies (BE0248, inVivoMab) and additionally treated with 20 ng/mL TGF-β for 72 h as previously described (Zundler et al., 2017c).

Subsequently, such cells were treated with a mouse anti-human β7 antibody (473207, R&D systems) or with or without etrolizumab-s at 37 or 4°C for 24 h. Where indicated, cells treated at 37°C were additionally permeabilized with 0.1% Triton X. Then, secondary staining was performed with goat anti-mouse antibodies and goat anti-rat antibodies labeled with the STED microscopy dye Star 580 (excitation: 594 nm pulsed laser, emission: 605–625 nm) or Star 635P, respectively (both Abberior, excitation: 640 nm pulsed laser, emission: 650–720 nm). Cell suspensions in Mowiol were covered on microscopy slides and analyzed with a STED microscope (Abberior 3D STED 2-Channel Super Resolution Microscope) equipped with a 100× Oil immersion lens (NA: 1.44). Stimulated emission depletion was performed at 775 nm with a pulsed laser. The power of the STED laser was set to 625 mW.

### Antibody-Dependent Cytotoxicity (ADCC) Assay With PBMCs

Antibody-dependent cytotoxicity assays were carried out using PBMCs from healthy donors as effector cells and the human lymphoma cell line WIL2-S (ATCC) as target cells. Target cells (4 × 10^4^) in 50 μL assay medium (RPMI-1640 with 1% BSA and 100 U/mL penicillin/streptomycin) were seeded in each well of a 96-well, round-bottom plate. Serial fourfold dilutions (1000 to 0.0038 ng/mL) of etrolizumab and the anti-CD20 antibody rituximab as a positive control (50 μL/well) were added to the plates containing the target cells, followed by incubation at 37°C for 30 min to allow opsonization. Subsequently, 10^6^ PBMC effector cells in 100 μL of assay medium were added to each well and the plates were incubated for an additional 4 h. After centrifugation, the supernatants were assayed for lactate dehydrogenase (LDH) activity using a Cytotoxicity Detection Kit (Roche Diagnostics). Cell lysis was quantified through absorbance at 490 nm using a microplate reader. Absorbance of wells containing only the target cells served as Low Control and wells containing target cells lysed with Triton-X100 as High Control. Antibody-independent cellular cytotoxicity (AICC) was measured in wells containing target and effector cells without the addition of antibody. The extent of specific ADCC was calculated as follows:

$$\%\;\mathrm{ADCC}\;=100\times (A_{490\mathrm{nm}}\left(\mathrm{High}\;\mathrm{Control}\right)-A_{490\mathrm{nm}}\left(\mathrm{Low}\;\mathrm{Control}\right))/(A_{490\mathrm{nm}}\left(\mathrm{Sample}\right)-A_{490\mathrm{nm}}\left(\mathrm{AICC}\right))$$

The mean ADCC values from duplicates were plotted against the antibody concentration, and the EC50 values were generated by fitting the data to a four-parameter equation with SoftMax Pro.

### Complement-Dependent Cytotoxicity (CDC) Assay

The CDC assays were carried out using WIL2-S cells as target cells and complement derived from human serum (Quidel Corporation). Etrolizumab and the anti-CD20 antibody rituximab were serially diluted in assay medium (RPMI-1640 supplemented with 20 mM HEPES pH 7.2, 0.1% BSA, and 0.1 mg/mL gentamicin), and distributed into a 96-well tissue culture plate (Costar; Corning Inc.). Following the addition of human serum complement (diluted 1:3 in assay medium) and the target cells (10^5^ cells/well), the plate was incubated 12 h at 37°C. After the incubation, AlamarBlue was added at 50 μL/well and the plate was incubated for an additional 15–18 h. The CDC of the test antibody was quantified through absorbance at 530 nm excitation with 590 nm emission on a fluorescence plate reader (SpectraMax GeminiXS, Molecular Devices). The EC50 values were generated by fitting the data to a four-parameter equation (SoftMax Pro).

### Induction of Pro-inflammatory Cytokine Production

Etrolizumab was evaluated *in vitro* both as a single agent and in the presence of a sub-stimulatory concentration of anti-human CD3 (4 ng/ml) for induction of pro-inflammatory cytokine/chemokine production in purified human PBMCs. PBMCs were assayed in 96-well microtiter plates (either with or without anti-CD3 coating) with trastuzumab, mouse IgG1, or etrolizumab, in solution phase. Positive control wells were coated with both anti-CD3 (4 ng/ml) and anti-CD28 (100 ng/ml), whereas negative control wells remained uncoated. Duplicate wells without anti-CD3 were also assayed, to determine the capability of the solution-phase mAbs to stimulate cytokine/chemokine production in PBMC without a costimulatory signal. Each well contained 4 × 10^5^ PBMC in 100 μL medium and plates were incubated at 37°C. Supernatants were collected following 24 and 48 h of culture and assayed by Luminex assays (Luminex Corp.) for cytokine and chemokine concentrations.

### Statistics

Unless otherwise stated, data are displayed as mean with error bars representing the standard error of the mean. Statistical comparisons were performed with Graph Pad Prism software (Graph Pad Software) applying one- or two-way ANOVA as applicable. *Post hoc* testing was done with Newman-Keuls or Bonferroni tests, respectively. Levels of significance are indicated by asterisks (^∗^*p* < 0.05, ^∗∗^*p* < 0.01, ^∗∗∗^*p* < 0.001).

## Results

### β7 Integrin Translocates From the Cell Surface to the Inside of the Cell Upon *in vitro* Treatment of Lymphocytes With Etrolizumab-s

**FIGURE 1:**
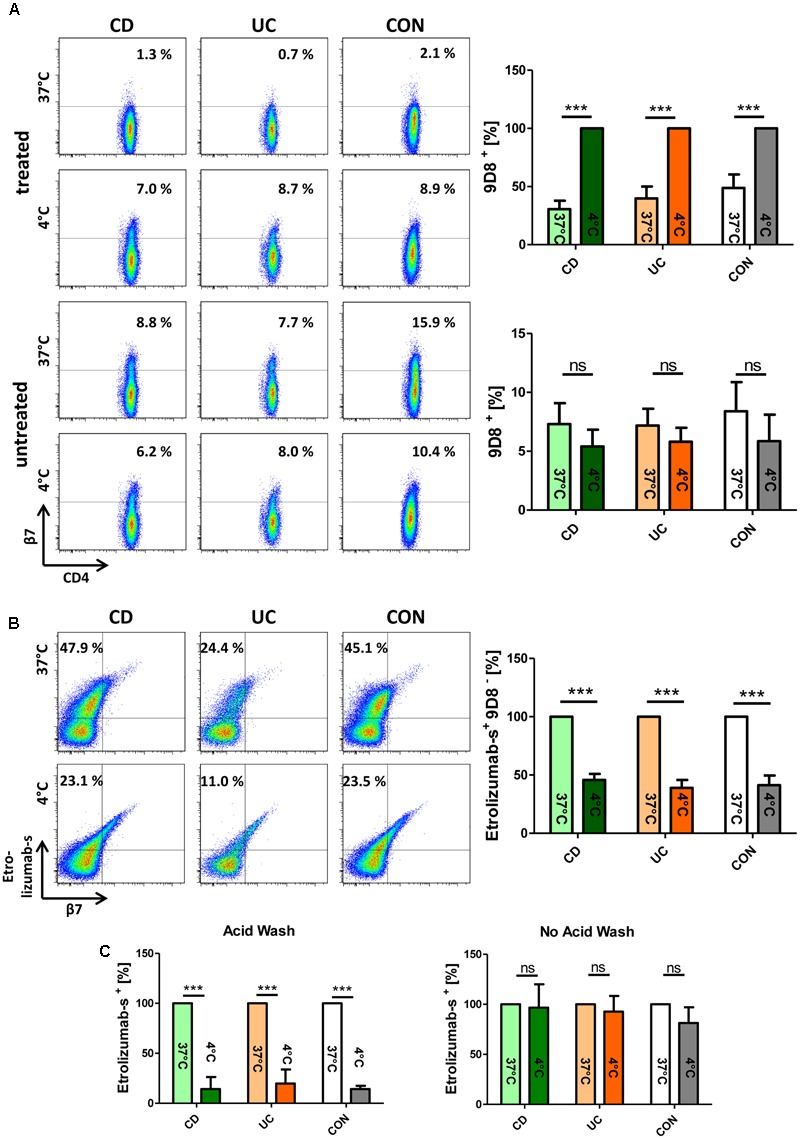
β7 integrin translocates from the cell surface to the inside of the cell upon *in vitro* treatment of lymphocytes with etrolizumab-s. **(A)** Upper panels: Flow cytometry of peripheral blood mononuclear cells (PBMCs) from patients with Crohn’s disease (CD), ulcerative colitis (UC), and control donors (CON) treated with etrolizumab-s for 24 h at 4 and 37°C. Left: Representative flow cytometry. The frequency of 9D8^+^ staining on CD4^+^ T cells is indicated. Right: Quantitative flow cytometry showing surface expression of 9D8 at 37°C relative to expression at 4°C (*n* = 5 per group). Lower panels: Representative (left) and quantitative flow cytometry (right) of cells cultured at 4 and 37°C without etrolizumab-s treatment. **(B)** Flow cytometry of PBMCs from CD, UC, and CON donors treated with AF-647-labeled etrolizumab-s. Left: Representative flow cytometry. The frequency of etrolizumab-s^+^9D8^-^ cells among CD4^+^ T cells is indicated. Right: Quantitative flow cytometry showing the proportion of these cells at 4°C in relation to 37°C (*n* = 5 per group). **(C)** Quantitative flow cytometry of PBMCs treated with AF647-labeled etrolizumab-s at 4 or 37°C and subsequently subjected to acid wash (left) or not (right). *n* = 5 per group; data are normalized to expression at 37°C. ^∗∗∗^*p* < 0.001; ns – not significant.

To assess whether β7 integrin is internalized upon etrolizumab treatment, we isolated PBMCs from the peripheral blood of healthy controls and IBD patients and incubated these cells with or without etrolizumab-s *in vitro*. This was performed at 37°C to allow internalization or at 4°C to prevent internalization (Wyant et al., 2013). After 24 h, cells were stained for flow cytometry with an antibody panel including the anti-β7 antibody 9D8 which recognizes β7 in the presence of etrolizumab-s through binding to a different epitope (Stefanich et al., 2011). At 4°C, as expected due to the absence of internalization, no difference in the abundance of CD4^+^9D8^+^ T cells could be observed between samples treated with or without etrolizumab-s (Figure 1A). At 37°C, however, the proportion of CD4^+^ T cells staining positive for 9D8 was significantly lower after etrolizumab-s treatment compared with no treatment in control donors as well as IBD patients with CD or UC. This suggested that the β7 integrin was reduced on the cell surface following incubation with etrolizumab-s.

To investigate the fate of β7 after such treatment, we performed an additional series of experiments, in which PBMCs were incubated with AF647-labeled etrolizumab-s and stained for flow cytometry after 24 h. We observed that the proportion of etrolizumab-s^+^9D8^-^ CD4 T cells was significantly higher after incubation at 37°C than at 4°C (Figure 1B). Therefore, we concluded that the fluorescence signal of etrolizumab-s must originate from the inside of the cells, since the 9D8 antibody was only able to bind surface-expressed β7 integrin. This was confirmed by another approach, in which we incubated PBMCs with AF647-labeled etrolizumab-s at 4 and 37°C for 24 h and applied an acid wash procedure afterwards to remove surface-bound antibody. Unsurprisingly, the substantial proportion of etrolizumab-s^+^ cells that could be observed without acid wash treatment at 4°C was almost completely lost, when acid wash was applied (Figure 1C). In contrast, a considerable amount of etrolizumab-s^+^ cells was observed at 37°C even when acid wash was performed, which similarly argued for an intracellular origin of the fluorescence signal. Thus, taken together, these results supported the conclusion that etrolizumab-s leads to internalization of β7 integrin.

### β7 Integrin Is Internalized Following Treatment of Lymphocytes With Etrolizumab

**FIGURE 2:**
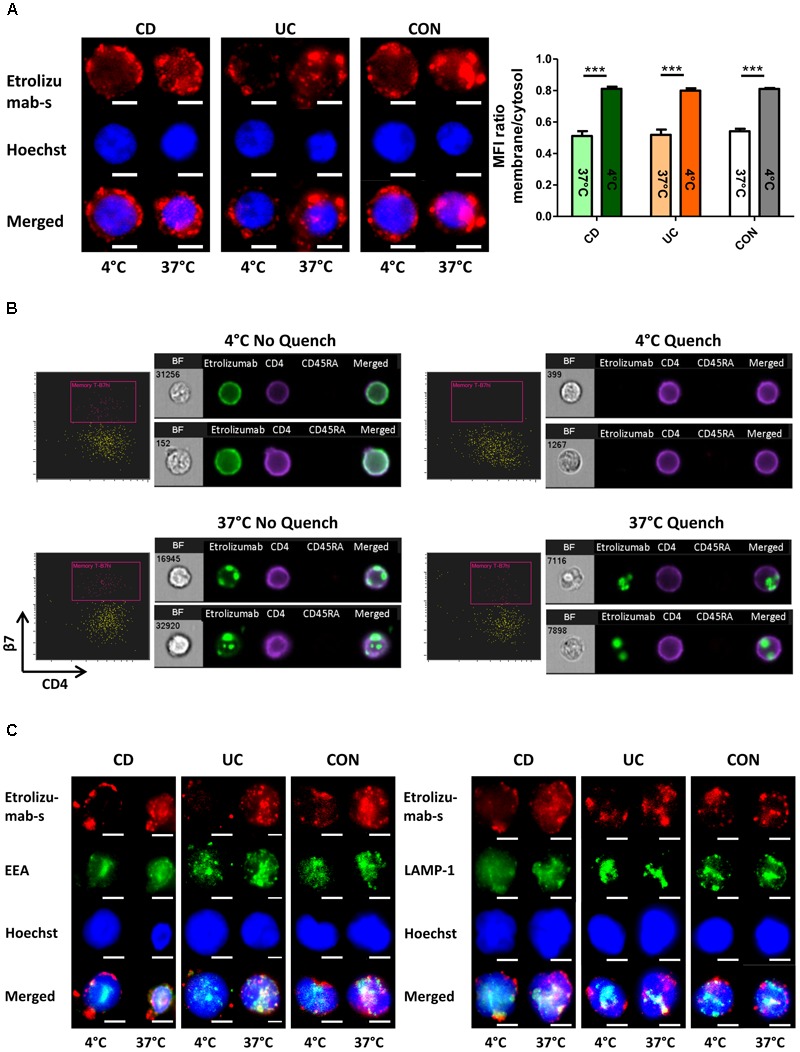
β7 integrin is internalized by treatment of lymphocytes with etrolizumab(-s). **(A)** Left: Representative images showing localization of the fluorescence signal of AF647-labeled etrolizumab-s after treatment of cells from CD, UC, and CON donors at 4 or 37°C for 24 h. Scale bar: 3 μm. Right: Quantification of mean fluorescence intensity (MFI) of AF647 signal in the cytosol relative to the membrane at 4 and 37°C; *n* = 5 per group. ^∗∗∗^*p* < 0.001. **(B)** Assessment of etrolizumab internalization with ImageStream^®^. Representative flow cytometry and microscopy results after cell incubation with AF488-labeled etrolizumab at 4°C (upper row) or 37°C (lower row) and without (left panels) or with (right panels) quench procedure. BF, bright field. Data are representative for two independent experiments with a total of eight samples. **(C)** Representative images showing localization of AF647 fluorescence signal of etrolizumab-s and AF488 fluorescence signal of EEA (left) and LAMP-1 (right) after cell treatment with etrolizumab-s at 4 or 37°C for 24 h. Scale bar = 3 μm. Images are representative for three independent experiments.

We therefore aimed to complementarily explore the internalization process with fluorescence microscopy. Accordingly, we treated PBMCs from the peripheral blood of controls and IBD patients with AF647-labeled etrolizumab-s at 4 and 37°C for 24 h. After counterstaining with Hoechst, cells were evaluated by fluorescence microscopy. Following incubation at 4°C, we observed a halo of etrolizumab-s fluorescence signal around the cell membrane in a portion of cells consistent with β7 expression on the cell surface (Figure 2A). In contrast, the signal observed in cells treated at 37°C was shifted to the inside of the cells. When quantifying the fluorescence signal in the latter compared with the former location by measuring the MFI for AF647, we found a highly significant difference in the ratio of surface and intracellular fluorescence intensity. Consistent with the flow cytometry results, these immunofluorescence images provided further evidence of β7 internalization after etrolizumab-s binding.

Subsequently, we explored whether these observations with etrolizumab-s also applied to the humanized therapeutic antibody etrolizumab. In an approach combining flow cytometry with fluorescence microscopy we analyzed internalization of β7 integrin following treatment with AF488-labeled etrolizumab at 4 and 37°C for 24 h with and without additional anti-AF488 quench (Figure 2B). When incubated at 4°C, as expected, superficial AF488 signal could be observed without, but not with quench procedure. At 37°C, however, microscopy demonstrated only minimal superficial AF488 staining that was removed by the quench procedure, but substantial intracellular signal that was not affected by quenching of AF488. Consistently, flow cytometry demonstrated AF488^+^ cells without and with quench at 37°C.

Additionally, we performed dual staining with AF647-labeled etrolizumab-s and the lysosome marker LAMP-1 or the endosome marker EEA to assess the intracellular location of etrolizumab-s after internalization (Figure 2C). At 37°C, but not at 4°C co-localization of both EEA-1 and LAMP-1 with etrolizumab-s could be observed suggesting internalization of the antigen-antibody complex into early endosomes and late endosomes/lysosomes, respectively.

**FIGURE 3:**
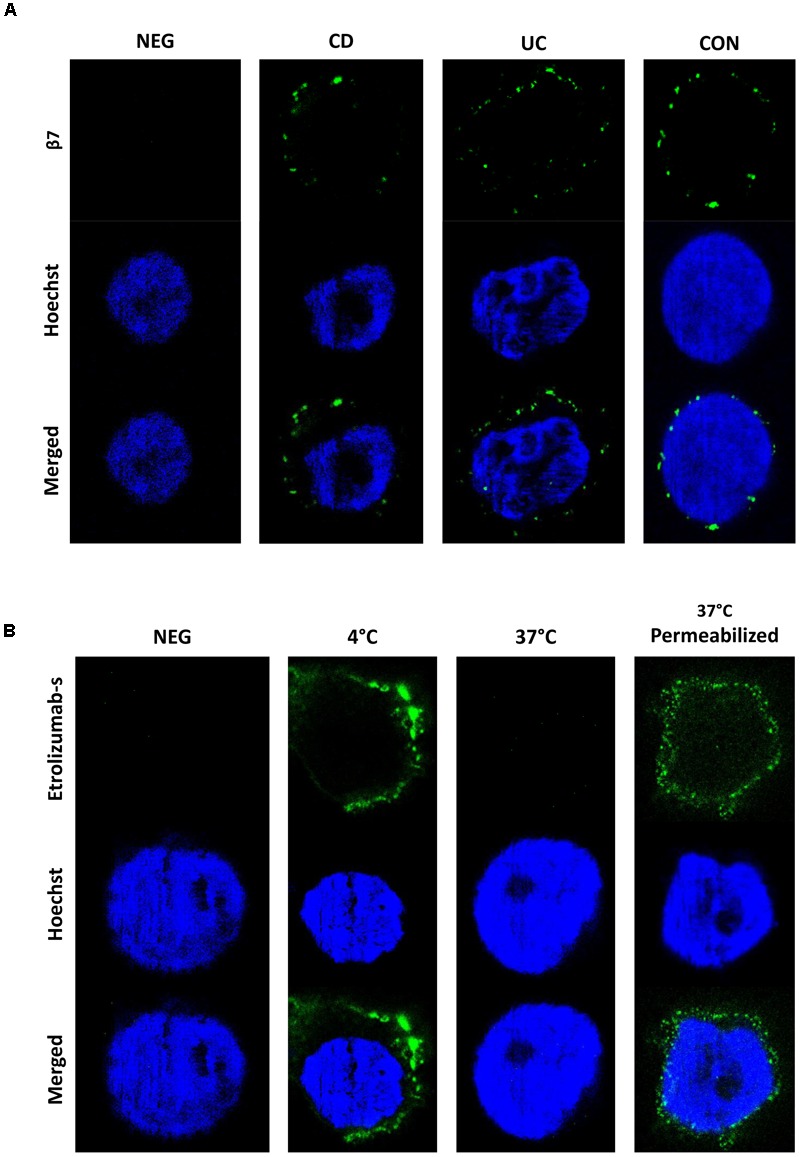
Assessment of etrolizumab-s internalization by STED microscopy. **(A)** Representative STED microscopy images showing localization and distribution of β7 integrin on the surface of cells from CD, UC, and CON donors. Additionally, a negative control (NEG) without primary antibody staining is shown. Images are representative for seven independent experiments. **(B)** Representative images showing etrolizumab-s localization on/in cells incubated with etrolizumab-s at 4°C or cells incubated with etrolizumab-s at 37°C and additionally treated with or without Triton-X. Additionally, a negative control without primary antibody staining is shown. Images are representative for five independent experiments.

To evaluate the internalization of β7 in even greater detail and on single-molecule level, we made use of STED microscopy, an innovative technology recently introduced to overcome the resolution limitations of conventional microscopic techniques (Blom and Widengren, 2017). Initially, we assessed the expression of β7 integrin induced by TGF-β on PBMCs from IBD patients and controls. Interestingly, β7 expression was not homogenously distributed around the cells, but confined to certain spots (Figure 3A). Subsequently, cells were treated with etrolizumab-s at 4 and 37°C for 24 h and secondary staining with Star635P-labeled anti-rat antibodies was performed. After treatment at 4°C, as anticipated, we observed a fraction of cells with a positive signal, which had a “halo” location consistent with cell surface expression of β7 integrin (Figure 3B). However, no such signal could be observed on cells treated at 37°C, presumably due to internalization of β7 and, thus, unavailability of etrolizumab-s on the cell surface for secondary staining. To directly demonstrate internalization, we additionally permeabilized cells with Triton-X prior to addition of the secondary antibody. Indeed, this treatment led to detection of a positive signal in projection to the nucleus or directly adjacent, therefore indicating internalized β7 integrin.

### Etrolizumab-Driven α4β7 Integrin Internalization Functionally Leads to Decreased Dynamic Adhesion to MAdCAM-1

**FIGURE 4:**
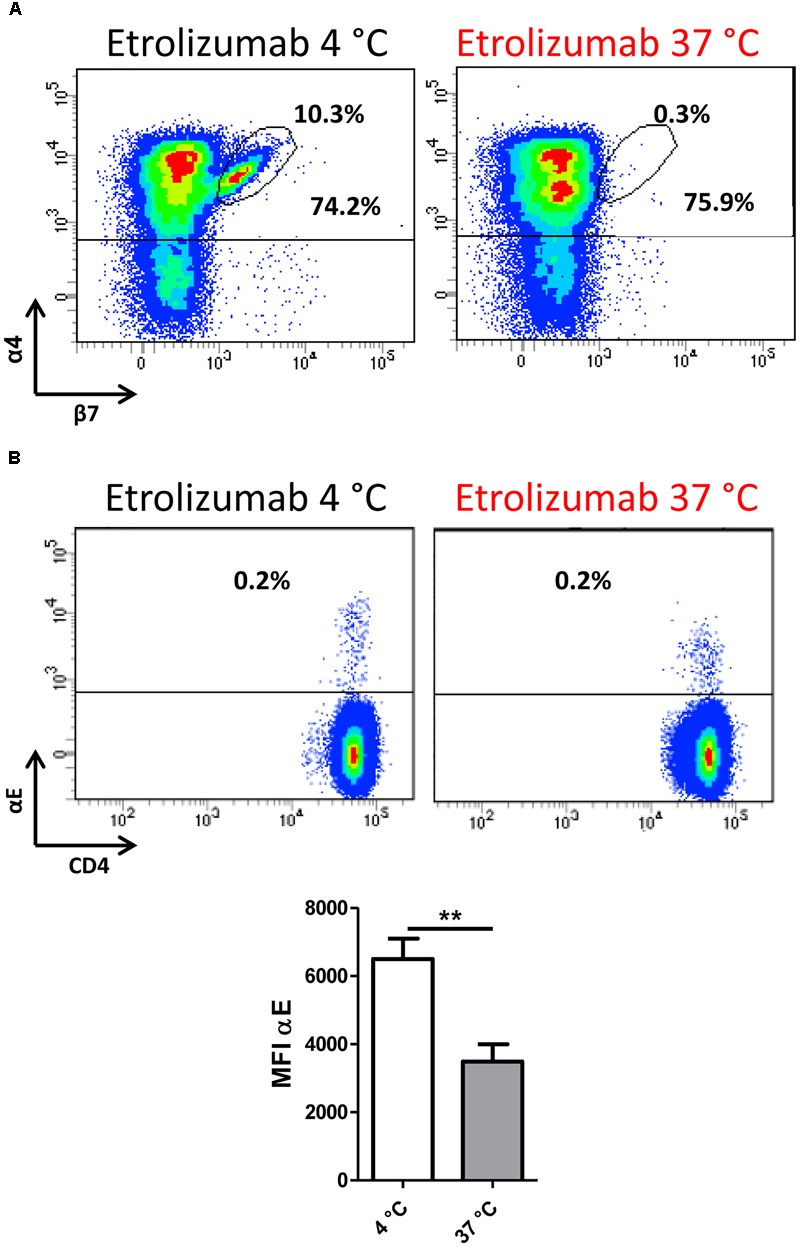
β7 internalization minimizes α4β7 expression on the cell surface. **(A)** Representative flow cytometry plot showing expression of α4β7 integrin after treatment with etrolizumab at 4 and 37°C. Percentages indicate α4^+^β7^+^ and total α4^+^ cells. **(B)** Upper panels: Representative flow cytometry plots showing expression of αE integrin after treatment with etrolizumab at 4 and 37°C. Lower panel: Quantification of β7 mean fluorescence intensity (MFI) of β7^+^ cells (*n* = 5). ^∗∗^*p* < 0.01.

Next, we sought to determine whether internalization of β7 following etrolizumab treatment equally affects internalization of the β7 integrin monomer paired with α4 or αE. As demonstrated by flow cytometry, we observed a loss of α4β7 following treatment with etrolizumab *in vitro* at 37°C compared with 4°C (Figure 4A), although the number of α4^+^ cells was not substantially affected. Similarly, the abundance of αE remained unchanged, although the MFI of αE^+^ cells significantly decreased after treatment at 37°C (Figure 4B). Together, these findings suggested that etrolizumab predominantly induces monomer internalization leading to absence of surface α4β7, and possibly also partial co-internalization of αE.

**FIGURE 5:**
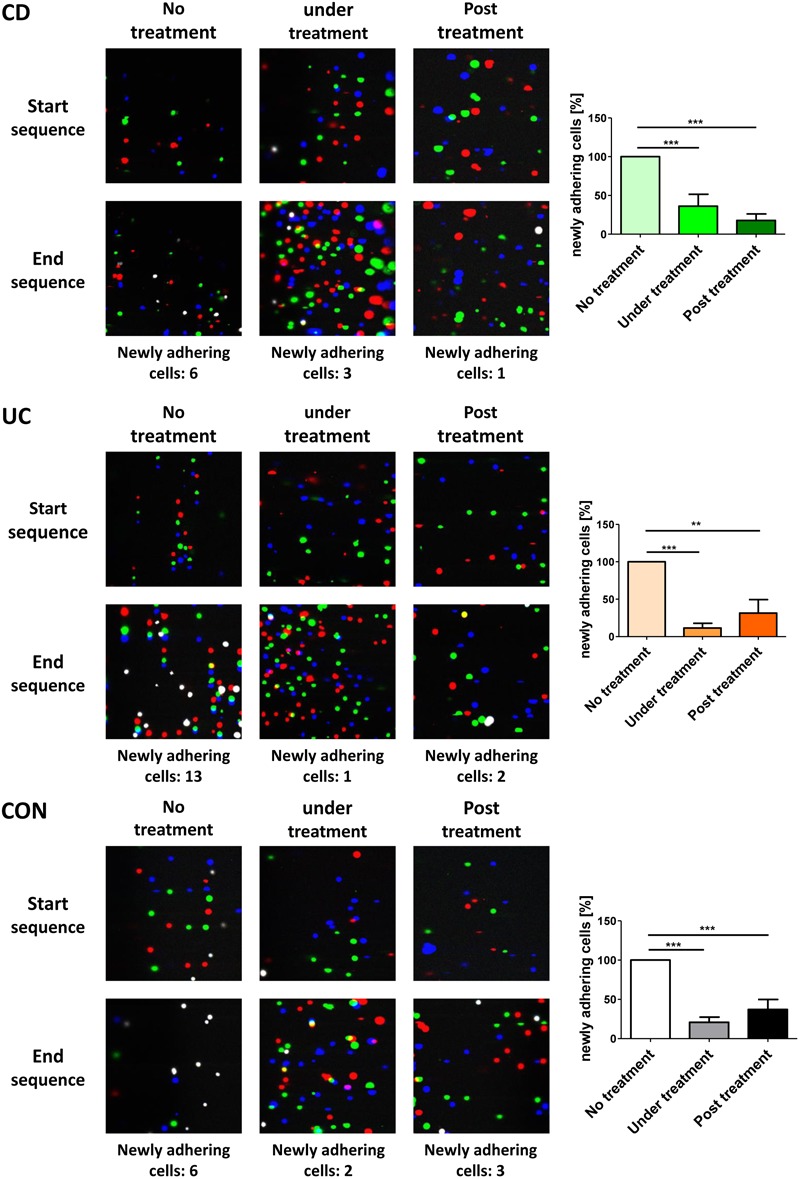
Internalization β7 integrin functionally leads to decreased dynamic adhesion to MAdCAM-1. Dynamic adhesion assays with untreated PBMCs, PBMCs pre-treated with etrolizumab-s for 24 h or treated with etrolizumab-s during the assay. Left panels: Representative overlays of three differentially colored sequential images collected at the beginning or end of 3 min clips. White cells represent adhering cells. Right panels: Quantitative data normalized to untreated PBMCs (CD *n* = 8, UC *n* = 5, and CON *n* = 5). ^∗∗^*p* < 0.01, ^∗∗∗^*p* < 0.001.

To investigate what α4β7 internalization functionally means for interaction with MAdCAM-1, we used a dynamic adhesion assay to study integrin-addressin interactions under shear stress (Binder et al., 2018). PBMCs were isolated from the peripheral blood of control donors and IBD patients and incubated with or without etrolizumab-s at 37°C for 24 h to induce internalization of β7 integrin or not, respectively. Subsequently, treated cells were extensively washed to remove any remaining antibody from the cell suspension. Previously untreated cells were either left untreated or treated with etrolizumab-s directly prior to perfusion through MAdCAM-1-coated ultrathin glass capillaries in the presence of etrolizumab-s. Substantial adhesion could be observed, when completely untreated cells were perfused. However, when cells treated with etrolizumab-s for 24 h and cells concomitantly treated with etrolizumab-s were used, dynamic adhesion was markedly and similarly reduced (Figure 5). Thus, internalization of β7 integrin following incubation with etrolizumab-s led to a decrease in dynamic adhesion to MAdCAM-1 even when no etrolizumab-s was present during perfusion and this effect was comparable to that observed with concomitant treatment. These results indicated that β7 integrin internalization induced by etrolizumab is functionally important for the impairment of interactions with MAdCAM-1.

### Cell Surface Expression of β7 Integrin Is Restored After Removal of Etrolizumab-s

**FIGURE 6:**
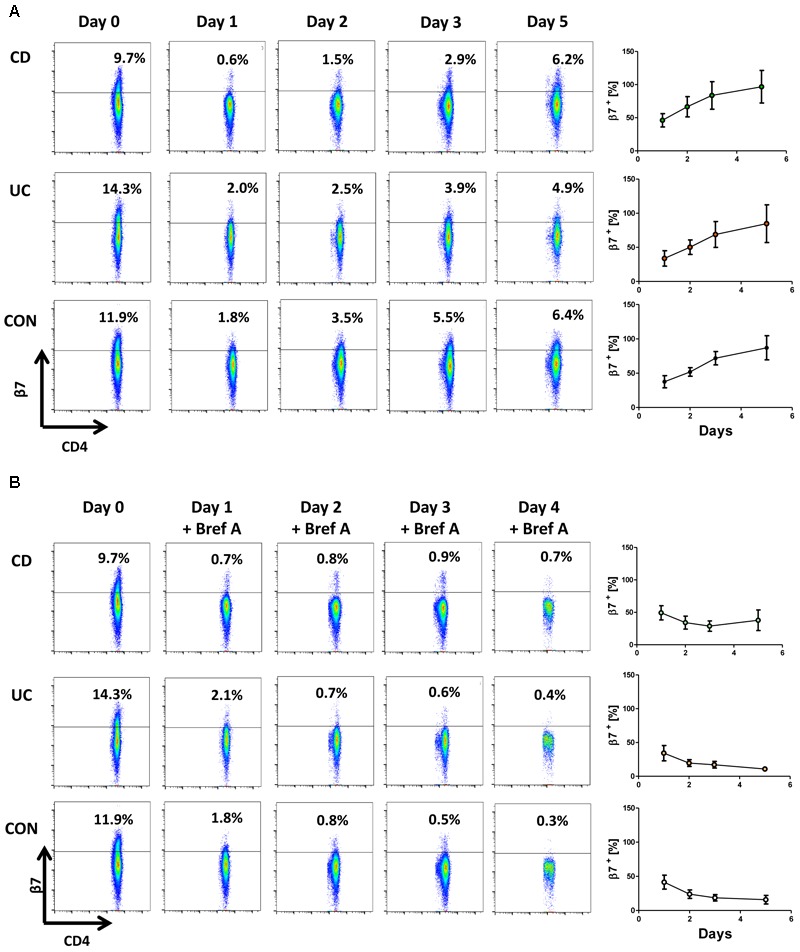
Cell surface expression of β7 integrin is restored after removal of etrolizumab-s. **(A)** Left panels: Representative flow cytometry showing β7 integrin expression at baseline (day 0) and 24 (day 1), 48 (day 2), 72 (day 3) and 120 h (day 5) after treatment with etrolizumab-s for the first 24 h of the experiment. Right panels: Quantitative flow cytometry of β7 surface expression over time relative to day 0 (*n* = 5–6 per group). **(B)** Left panels: Representative flow cytometry showing β7 integrin expression at baseline (day 0), and 24 (day 1), 48 (day 2), 72 (day 3) and 120 h (day 5) after treatment with etrolizumab-s for the first 24 h of the experiment and treatment with Brefeldin A from day 1 to day 5. Right panels: Quantitative flow cytometry of β7 surface expression over time relative to day 0 (*n* = 5–6 per group).

We then assessed whether β7 integrin is re-expressed on the cell surface after treatment with etrolizumab-s. To analyze this, PBMCs from the peripheral blood were incubated with etrolizumab-s for 24 h and aliquots of the cells were used to determine β7 integrin expression on CD4^+^ T cells with the 9D8 antibody (labeled with AF488) before and after treatment to confirm downregulation of surface β7 expression (Figure 6A). Subsequently, cells were washed to remove excess etrolizumab-s and further cultured for additional 96 h. Cell surface expression of β7 integrin was analyzed after 24, 48, and 96 h and was found to gradually increase until almost reaching pre-treatment levels after 4 days.

To address whether this was due to recycling of internalized β7 integrin or resulting from *de novo* synthesis, we performed an additional series of experiments, in which etrolizumab-s treatment was followed by application of Brefeldin A to inhibit Golgi transport of freshly translated β7 protein. During such incubation, surface β7 integrin expression persisted on the levels observed directly after treatment with etrolizumab-s (Figure 6B). Together, these results suggested that internalized β7-etrolizumab-s aggregates are degraded and β7 expression on the cell surface after removal of etrolizumab-s is restored due to *de novo*-synthesis of β7 integrin.

### Etrolizumab-s Does Not Elicit Agonistic Activity

**FIGURE 7:**
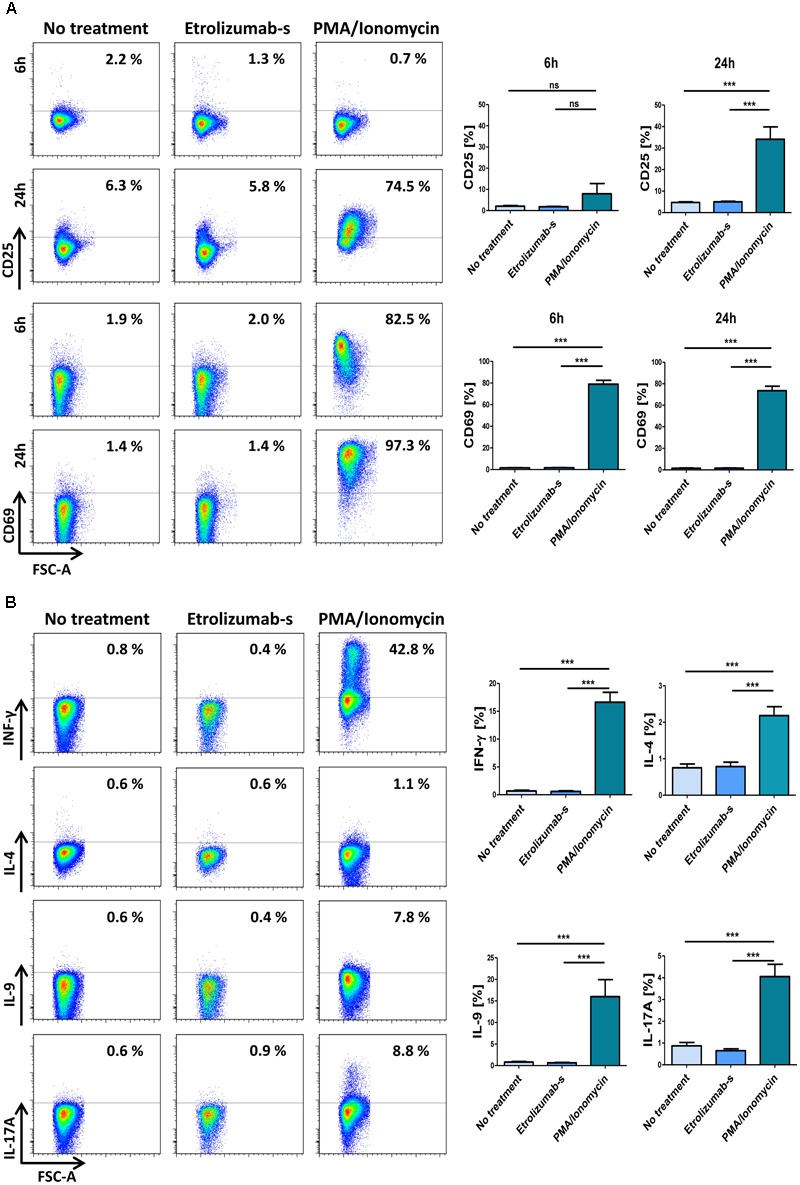
Etrolizumab-s does not elicit agonistic activity and cytokine production. **(A)** Left panels: Representative flow cytometry showing expression of CD25 (upper panels) and CD69 (lower panels) after cell treatment with or without PMA/ionomycin and etrolizumab-s for 6 or 24 h. Right panels: Quantitative flow cytometry (*n* = 11–26). **(B)** Left panels: Representative flow cytometry showing expression of pro-inflammatory cytokines after cell treatment with or without PMA/ionomycin and etrolizumab-s for 6 h. Right panels: Quantitative flow cytometry (*n* = 21–23 per group). ^∗∗∗^*p* < 0.001; ns – not significant.

Since monoclonal therapeutic antibodies can potentially have agonistic activity (Winkler et al., 1999), we addressed the expression of activation markers following incubation with etrolizumab-s for six and 24 h. Cells treated with PMA and ionomycin served as positive, untreated cells as negative control. As expected, stimulation with PMA/ionomycin caused a clear upregulation of CD69 on CD4^+^ T cells after six and 24 h (Figure 7A). In contrast, and in accordance with the literature (Wyant et al., 2013), CD25 was only modestly upregulated after six, but markedly increased after 24 h. No induction of CD69 or CD25 could be observed in untreated samples and, similarly, the expression in etrolizumab-s-treated samples was unchanged. These data indicated that binding of etrolizumab-s to β7 integrin does not lead to cell activation.

In a next step, we investigated whether etrolizumab-s has a direct effect on the expression of pro-inflammatory cytokines. Cells were left untreated or treated with etrolizumab-s or PMA/ionomycin for 2 h. Subsequently, Brefeldin A was added for additional 4 h and the expression of cytokines was assessed by flow cytometry. While increased expression of IFN-γ, IL-4, IL-9, and IL-17A could be observed in PMA/ionomycin-treated CD4^+^ T cells (Figure 7B), only very low and comparable production was found in untreated cells and etrolizumab-s-treated cells.

**FIGURE 8:**
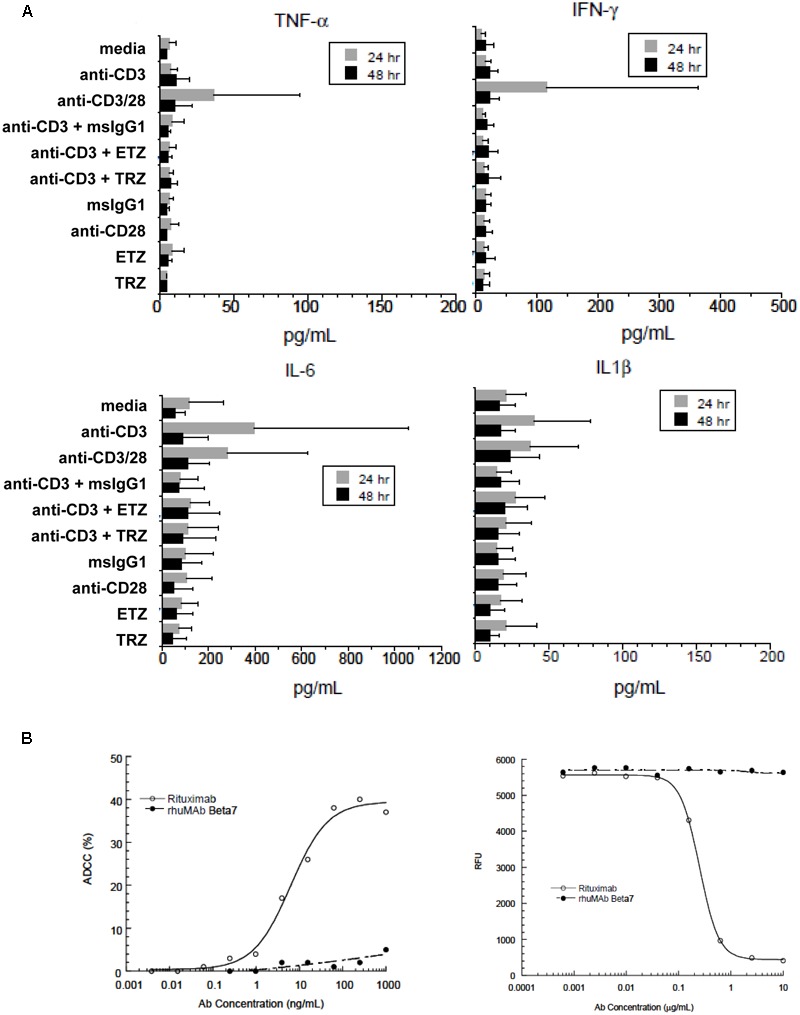
Etrolizumab does not elicit cytokine production and cytotoxicity. **(A)** Concentration of cytokines in supernatants of human PBMCs incubated with different antibodies with or without stimulation with anti-CD3 after 24 and 48 h. *n* = 6, mean +/– SD. **(B)** Left panel: ADCC activity of different concentrations of etrolizumab (rhuMAb Beta7) and rituximab in WIL2-S cells quantified as specified in Methods. Right panel: CDC assay with different concentrations of etrolizumab and rituximab in WIL2-S cells as specified in Methods. Relative fluorescence units (RFU) indicate the number of viable cells. Data are representative for three independent experiments.

Additionally, we assessed the concentration of several cytokines and chemokines in supernatants of PBMCs following treatment with etrolizumab (Figure 8A and Supplementary Figure S1). Consistently, cytokine and chemokine levels observed after etrolizumab treatment were similar to negative controls.

As monoclonal antibodies may also induce antibody-dependent cell-mediated cytotoxicity (ADCC) or complement-dependent cytotoxicity (CDC), we performed ADCC and CDC assays with etrolizumab using the human lymphoma cell line WIL2-S. The anti-CD20 antibody rituximab served as positive control. While substantial ADCC and CDC were observed with rituximab, neither was observed with etrolizumab (Figure 8B).

### Etrolizumab Is More Effective Than Vedolizumab in Inducing β7 Internalization

The anti-α4β7 antibody vedolizumab and etrolizumab both target the α4β7 integrin and internalization of α4β7 integrin in response to vedolizumab incubation has previously been described (Wyant et al., 2013). Therefore, we aimed to compare the efficacy of both compounds in inducing β7 integrin internalization.

**FIGURE 9:**
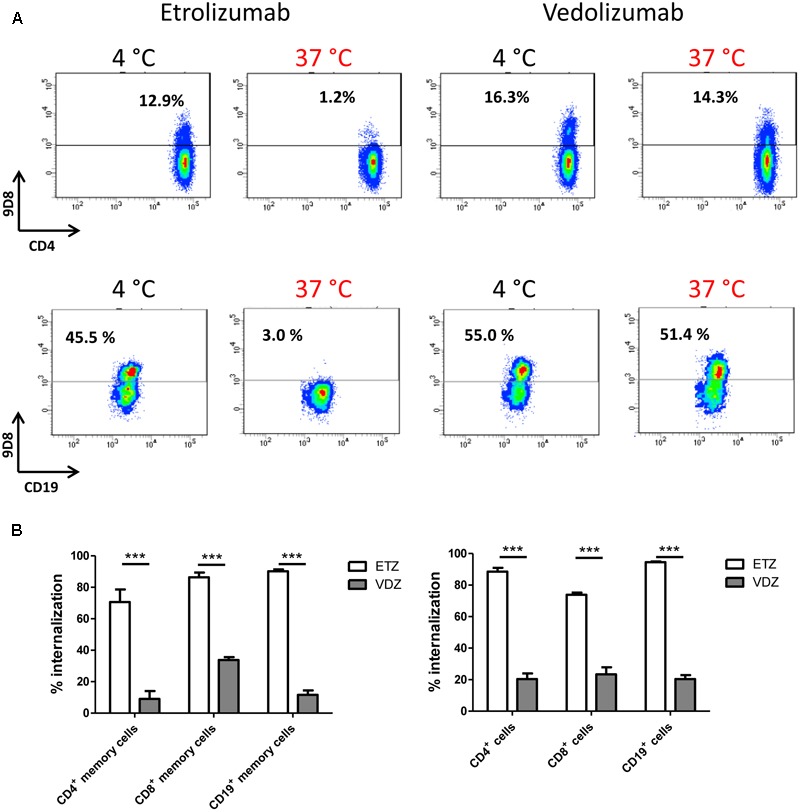
Comparison of β7 internalization after treatment with vedolizumab and etrolizumab *in vitro*. **(A)** Internalization of β7 on CD4^+^ T and CD19^+^ B cells from the peripheral blood of human donors. Representative dot plots indicating the percentage of β7^+^ cells upon treatment with etrolizumab or vedolizumab at 4 or 37°C as indicated. **(B)** Quantification of β7^+^ internalization (decrease of surface β7 expression related to expression observed after treatment at 4°C) in T and B cell subsets treated with etrolizumab or vedolizumab as indicated. Memory cells were defined as CD45RA^-^. All analyses were performed on β7^high^ cells (*n* = 5 donors). ETZ, etrolizumab; VDZ, vedolizumab; *n* = 10. ^∗∗∗^*p* < 0.001.

To this end, PBMCs were treated with vedolizumab or etrolizumab at 4°C and 37°C and surface β7 expression was analyzed in T and B cells. Consistent with our previous findings, etrolizumab treatment led to a clear reduction of β7 on all subsets studied (Figures 9A,B). Vedolizumab treatment also led to a decrease in expression and fluorescence intensity of surface β7, but this was clearly less marked than the decrease seen after etrolizumab treatment. Importantly, vedolizumab almost completely prevented binding of Act-1 (Supplementary Figure S2) indicating full saturation of α4β7 integrin.

## Discussion

Anti-integrin therapy has successfully been established as a new concept in the treatment of IBDs (Lobatón et al., 2014; Zundler et al., 2017a). Mechanistically, it is believed that targeted inhibition of the interaction of integrins with respective ligands by monoclonal antibodies leads to impaired cell trafficking and subsequent reduction of pro-inflammatory cell infiltration in the gut (Habtezion et al., 2016; Zundler and Neurath, 2017). However, beyond such mechanisms on the tissue level and regarding cell trafficking, the mode of action of these anti-integrin agents on their integrin target and the cellular consequences of antibody targeting are only partly understood. Here, we investigated the fate of β7 integrin upon binding of both etrolizumab-s and etrolizumab, which is currently being investigated in phase III studies in IBD. Etrolizumab is directly derived from the etrolizumab surrogate rat antibody FIB504 (Andrew et al., 1994) that was used in a part of the studies and has comparable binding properties (Stefanich et al., 2011). Consistently, the effects observed with etrolizumab-s paralleled the effects of etrolizumab, the humanized antibody.

On a single cell level, antibody binding to a target molecule may induce several cellular effects such as induction or blockage of cell activation and consecutive cytokine release. A prominent example for this effect is the anti-CD28 antibody TGN1412, which caused a cytokine storm with severe clinical consequences in participants of a phase I study (Suntharalingam et al., 2006). Increased cytokine secretion has also been observed in patients treated with the anti-CD20 antibody rituximab (Winkler et al., 1999), particularly in individuals with high peripheral cell numbers, and following treatment with the anti-CD3 antibody OKT3 (Gaston et al., 1991). Regarding etrolizumab, our *in vitro* data demonstrate that it does not cause upregulation of cell activation markers like CD25 and CD69, and that cytokine secretion by CD4^+^ T cells is also unaffected. Thus, agonist activity of etrolizumab on cell activation is highly unlikely. It is important to mention that these observations do not exclude effects of etrolizumab on cytokine expression on the tissue level as suggested by the decline reported in previous studies (Vermeire et al., 2014; Tew et al., 2016). This might be a secondary effect due to altered cell trafficking and reduced numbers of pro-inflammatory cells in the tissue.

Another potential mechanism mediated by therapeutic antibodies is the induction of ADCC or CDC as observed with the above mentioned antibodies rituximab and OKT3 (Raasveld et al., 1993; Golay et al., 2002). Both effects are mediated by the Fc fragment of the antibodies (Vidarsson et al., 2014). Our data suggest that etrolizumab does not elicit cytotoxicity via either mechanism. The observations reported in preclinical models and clinical studies, where the numbers of peripheral blood lymphocytes were stable or even increasing (Stefanich et al., 2011; Vermeire et al., 2014) under treatment, indicate that this is also not the case *in vivo*.

Internalization of the antibody-antigen complex into the cell is frequently observed in response to antibody treatment (Harper et al., 2013; de Goeij et al., 2014; Xu, 2015). We therefore hypothesized that this might also be true for etrolizumab. Indeed, our microscopic and flow cytometric data strongly support the conclusion that this is case, since, upon treatment with etrolizumab or etrolizumab-s, β7 could be detected intracellularly and reduced presence of β7 integrin on the cell surface was observed. This was finally confirmed by molecular microscopy using STED imaging, where β7 integrin vanished from the cell surface after etrolizumab-s treatment, but could be detected inside the cells after permeabilization. Thus, internalization seems to be a common feature of integrin ligation by neutralizing antibodies and even natural ligands. E.g., a similar mechanism has been reported for the anti-α4β7 antibody vedolizumab (Wyant et al., 2013) and the anti-rat α4 antibody TA2 (Leone et al., 2003). Moreover, it has been shown that engagement of α4β1 with its endothelial ligand vascular adhesion molecule (VCAM)-1 leads to internalization of the addressin (Ricard et al., 1998).

Using dynamic adhesion assays, we could additionally demonstrate that β7 internalization upon etrolizumab-s treatment has functional consequences and decreases adhesion to MAdCAM-1. Thus, receptor internalization might in fact explain the pre-clinical and clinical effects of etrolizumab (Stefanich et al., 2011; Vermeire et al., 2014).

However, compared to the *in vitro* results, the *in vivo* situation seems to be more complex. As demonstrated by the recent phase II trial with etrolizumab in UC (Vermeire et al., 2014), there was no apparent loss of β7 expression on the cell surface relative to baseline. Our data show that after removal of etrolizumab-s from cell cultures, β7 integrin is newly expressed within few days. Thus, surface-expressed β7 in etrolizumab-treated patients might at least partly result from *de novo* synthesis of the integrin, as it is highly likely that internalization and re-expression occur in parallel *in vivo*. In the light of recent studies suggesting compensatory mechanisms in response to anti-integrin therapies (Fuchs et al., 2017; Zundler et al., 2017b), it is also tempting to speculate that anti-integrin antibody treatment might trigger a compensatory increase in *de novo* integrin expression. Moreover, it has to be taken into account that anti-β7 treatment is believed to lead to an increased number of target cells in the peripheral blood due to impairment of gut homing (Vermeire et al., 2014; Binder et al., 2018). Thus, in synopsis, in addition to blockade, it is likely that internalization is one of several mechanisms of actions of etrolizumab *in vitro* and *in vivo*.

Our data are also in line with and a potential explanation for the favorable safety profile observed with etrolizumab and gut-specific anti-adhesion therapies in general so far (Lin et al., 2015; Luthra et al., 2015), since they suggest the absence of effector properties affecting key cellular functions other than β7 blockade (Liu et al., 2008).

In a final series of experiments, we compared the efficacy of β7 internalization after treatment with vedolizumab and etrolizumab *in vitro*. Our data indicate that etrolizumab is more effective in this regard and, thus, suggest that these anti-integrin antibodies do not only differ in regard to the overall mechanism of action (Zundler et al., 2017c), but may also act differently on a cellular level. E.g., it seems possible that different mechanisms of endocytosis apply. Although functional short-term effects on α4β7-dependent adhesion to MAdCAM-1 were similar for both antibodies (Binder et al., 2018), differential internalization properties might lead to differences in the efficacy of α4β7 blockade in the longer term.

Taken together, our data suggest that antibody-antigen complex internalization may be an important mechanism of action of etrolizumab and might help us better understand the clinical effects and the safety profile of etrolizumab in IBD.

## Author Contributions

CL, SK, EB, FF, HC, CR, and SC performed the experiments. RA, EK, CN, IA, MN, and SZ provided clinical samples, protocols, reagents, or designed the experiments. CL, SK, EB, FF, RE, SC, JM, MN, and SZ analyzed and interpreted the data. CL and SZ drafted the manuscript. All authors critically revised the manuscript for important intellectual content.

## Conflict of Interest Statement

SK, FF, RE, CR, HC, SC, and JM are employees of Genentech Inc. MN has served as an advisor for Pentax, Giuliani, MSD, Abbvie, Janssen, Takeda, and Boehringer. SZ and MN received research support from Takeda and Hoffmann-La Roche. The remaining authors declare that the research was conducted in the absence of any commercial or financial relationships that could be construed as a potential conflict of interest.

## Supplementary Material

FIGURE S1 Concentration of cytokines and chemokines in supernatants of human PBMCs incubated with different antibodies with or without stimulation with anti-CD3 after 24 and 48 h.

FIGURE S2 Incubation with vedolizumab hinders binding of Act-1. Representative dot plots showing binding of AF647-labeled Act-1 to PBMCs treated with (left) or without (right) vedolizumab.
