# Supplementary figures and images for: Cellular Mechanisms of Etrolizumab Treatment in Inflammatory Bowel Disease
Source: Front Pharmacol. 2019 Feb 1;10:39. doi: 10.3389/fphar.2019.00039 (PMC6367223; doi:10.3389/fphar.2019.00039)

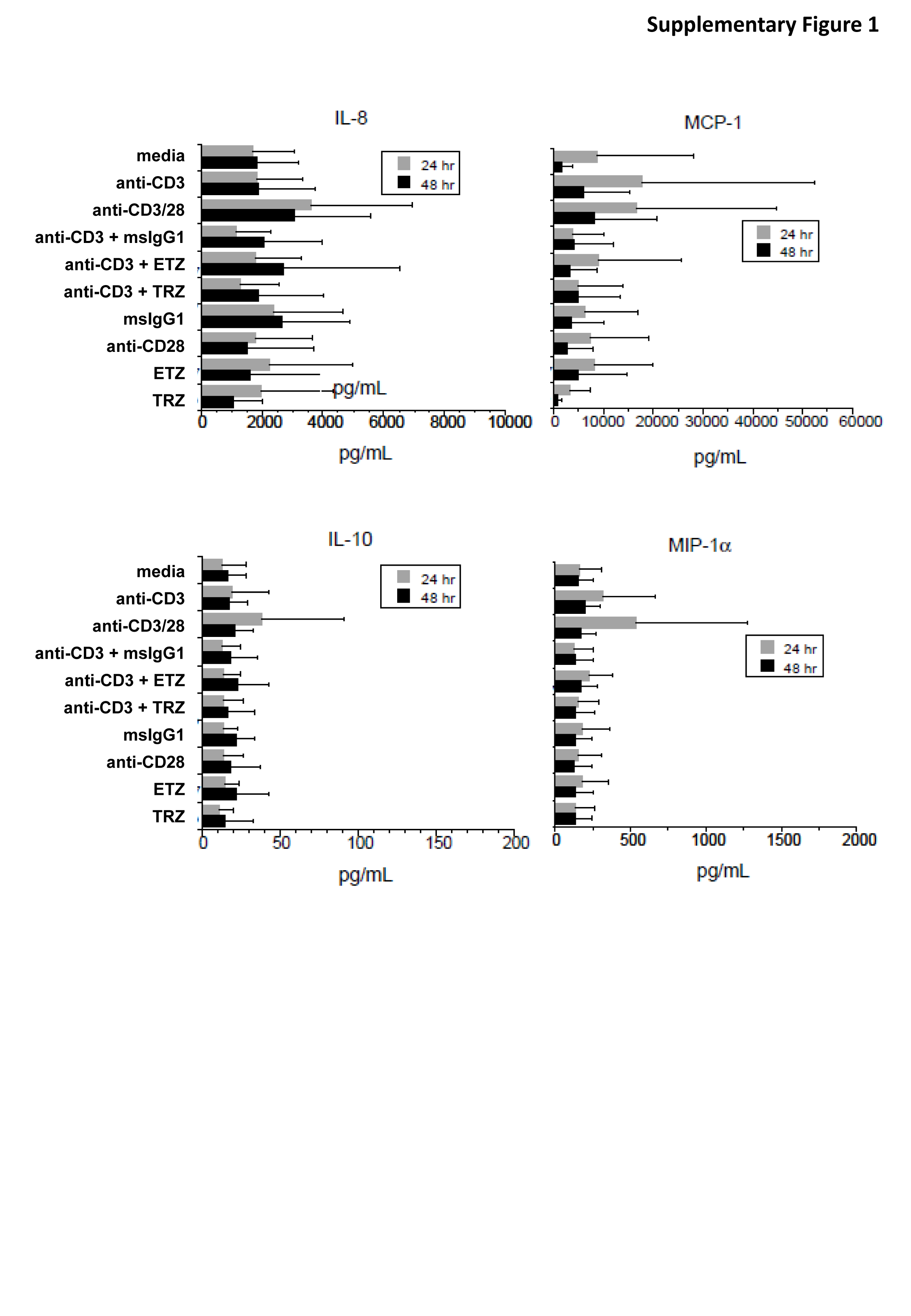

Supplement: FIGURE S1 — Concentration of cytokines and chemokines in supernatants of human PBMCs incubated with different antibodies with or without stimulation with anti-CD3 after 24 and 48 h. [file Image_1.TIF]

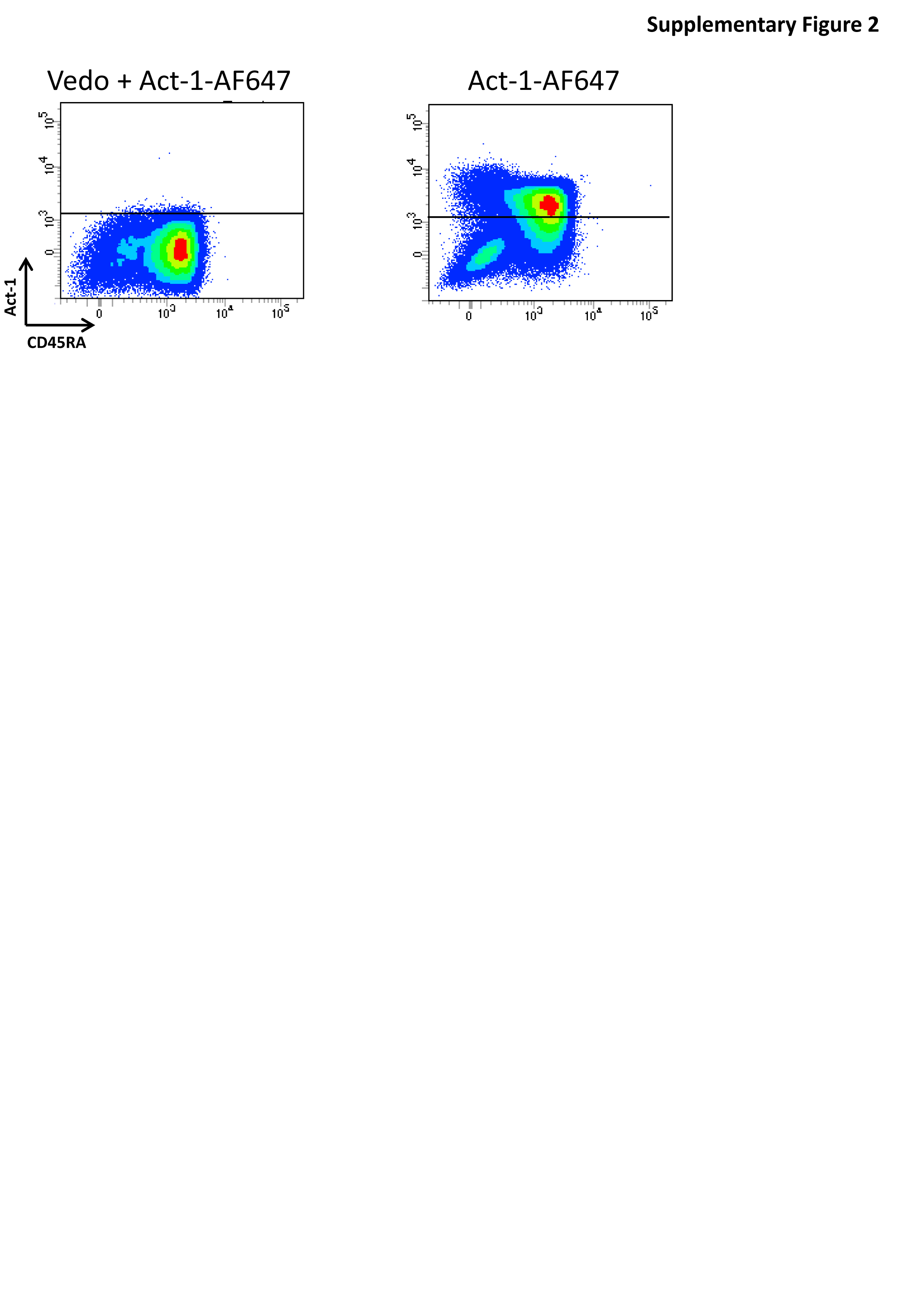

Supplement: FIGURE S2 — Incubation with vedolizumab hinders binding of Act-1. Representative dot plots showing binding of AF647-labeled Act-1 to PBMCs treated with (left) or without (right) vedolizumab. [file Image_2.TIF]
